# Coronary Plaque in Athletes

**DOI:** 10.3390/jcm13072044

**Published:** 2024-04-01

**Authors:** Elisabetta Tonet, Matteo Arzenton, Marco De Pietri, Luca Canovi, Davide Lapolla, Alberto Sarti, Veronica Amantea, Andrea Raisi, Gianni Mazzoni, Gianluca Campo, Giovanni Grazzi

**Affiliations:** 1 Cardiology Unit, Azienda Ospedaliero Universitaria of Ferrara, Via Aldo Moro 8, 44124 Ferrara, Italy; matteoarze@gmail.com (M.A.); marco.depietri1@gmail.com (M.D.P.); lucacanov@gmail.com (L.C.); davidelapolla@gmail.com (D.L.); sarticchi@gmail.com (A.S.); veronica.amantea@gmail.com (V.A.); cmpglc@unife.it (G.C.); 2 Center for Exercise Science and Sport, University of Ferrara, 44123 Ferrara, Italy; andrea.raisi@unife.it (A.R.); gianni.mazzoni@unife.it (G.M.); giovanni.grazzi@unife.it (G.G.)

**Keywords:** athletes, coronary artery disease, atherosclerosis

## Abstract

The relationship between vigorous physical activity (PA) and the development of coronary atherosclerosis has remained less explored for many years. Recently, literature data have focused on coronary atherosclerosis in athletes showing that prevalence is not trivial, that there are differences among various types of sport, and that there are some peculiar features. As a matter of fact, plaque composition in athletes seems to be characterized by calcium rather than soft components. Specific mechanisms through which vigorous PA influences coronary artery disease are not yet fully understood. However, the prevalent calcific nature of coronary plaques in athletes could be related with a trend in a lower cardiovascular event rate.

## 1. Introduction

Atherosclerotic Coronary Artery Disease (CAD) is a progressive disease that affects a substantial part of the population by middle age, and it is often subclinical, becoming more likely to be evident over time. Coronary artery plaques are typically associated with sedentary lifestyles, poor dietary habits, and common risk factors including overweight/obesity and smoking/tobacco use.

Physical inactivity is an additional risk factor for CAD, and conversely, regular physical training reduces the risk of developing CAD [1]. However, participation in competition events increases the level of stress, and adherence to prolonged high-to-very-high-intensity exercise training schedule is a major challenge. CAD is the most prevalent cause of exercise-related cardiac events in individuals with established coronary syndrome, or sudden cardiac death as a primary presentation in individuals > 35 years of age [2].

Adult athletes, variously defined as people older than age 30, 35, or 40, are commonly engaged in prolonged and vigorous training regimens, and they often experience remarkable favorable cardiovascular adaptations. However, there is evidence that they are not immune to the development of coronary artery plaques [1]. This phenomenon challenges our understanding of how exercise impacts coronary health and underlines the importance of comprehensive cardiovascular assessment in athletes. As a matter of fact, understanding the unique challenges and manifestations of CAD in athletes is crucial for early detection, and an appropriate management strategy, as it not only affects the individual athlete’s health but also has significant implications for their performance and overall well-being. In the present review, we explore the intriguing relationship between athletes and coronary artery plaques, shedding light on epidemiology, the potential factors contributing to this unexpected occurrence, plaque composition, and finally plaque progression and its relationship with cardiovascular events in athletes.

## 2. Epidemiology

The relationship between physical activity (PA) and the development of coronary atherosclerosis has remained less explored for many years. Recently, literature data have focused on coronary atherosclerosis in athletes for two reasons: (a) the number of master athletes is increasing, and (b) adverse events related to coronary atherosclerosis represent the main cause of exercise-related death in athletes over 35 years old [1,2,3]. Initial studies on atherosclerosis and vigorous exercise intensity highlighted that PA was associated with a higher amount of coronary artery calcium (CAC) [4,5].

A study by Mohlenkamp et al. demonstrated that in a population of athletes, the use of traditional calculators could underestimate cardiovascular risk. As a matter of fact, CAC values of runners over 50 years old were similar to those of age-matched controls but higher when adjusted for risk factors [6]. In 2016, Braber and colleagues estimated the prevalence of CAD using CAC score and coronary computed tomography angiography (CCTA) in a population of 318 sportsmen over 45 years old with a low-risk profile according to the Systematic Coronary Risk Estimation (SCORE) tool and normal exercise tests. They demonstrated how the combination of these two tests could reveal occult CAD in 1 out of 5 subjects, despite negative first-level tests [7]. Aengevaeren V.L. et al. correlated type, intensity, and duration of PA with CAC score values and the type of atherosclerotic plaques identified by CCTA. The analysis showed that more intensive and prolonged exercise training expressed as metabolic equivalent (MET)-minutes/week was associated with higher CAC score values and a higher incidence of calcific plaques compared to mixed ones, which are more frequent in less-trained subjects [8].

These findings were confirmed by Merghani’s study in 2017 [9]. In a population of 152 master athletes compared with controls of similar age, sex, and low Framingham risk score, there were no differences in the absolute prevalence of CAC. Approximately 60% of both categories had CAC = 0, and when present, the coronary calcium of the athletes once again was higher in terms of CAC score values when compared to controls. Even though the number of atherosclerotic plaques was higher in the athletes’ group, plaques were more calcific and, therefore, probably less dangerous than the fibro-lipidic ones.

Regarding specific plaque composition and morphology in athletes, a tendency toward an increased occurrence of coronary atherosclerotic plaques among lifelong endurance athletes when compared to healthy non-athletes has been reported in several studies [6,7]. Other studies have focused on the prevalence and composition of coronary plaques based on the type of sport. In this context, male runners compared to sedentary controls showed an increased total plaque volume [10]. It was also proved that cyclists exhibited a reduced prevalence of atherosclerotic plaques and a lower prevalence of CAC compared to runners [11,12]. Regarding sex differences, data are lacking among women, who are known to have a lower probability of coronary atherosclerosis and risk of exercise-related cardiac arrest than men [13]. At present, only two studies with cohorts of fewer than 100 female individuals have investigated the prevalence of coronary atherosclerosis between athletes and controls. In the first one, no significant difference was found, neither in terms of the prevalence of coronary plaques, nor in terms of their composition [9]. In the second one, only marathon runners were included, and female marathon runners exhibited minimal counts of CAC, a lower prevalence of coronary artery plaque, and reduced calcified plaque volume compared to inactive women [14].

## 3. Coronary Plaque Composition in Athletes

Coronary plaques can be classified into calcified, non-calcified, and mixed plaques according to their composition. Understanding features, composition, and volume of atherosclerotic plaques is relevant in terms of prognosis in the context of athletes. A more challenging prognosis is associated with mixed and non-calcified plaques due to their intrinsic vulnerability and lipid-rich composition, while calcified plaques tend to be considered more stable and less prone to rupture [15].

CCTA allows anatomical characterization of coronary atherosclerosis and assessment of the CAC score. The latter strongly correlates with the overall burden of coronary plaques and independently predicts cardiovascular events, regardless of age. A CAC score of zero holds a high negative predictive value for ruling out significant coronary atherosclerotic disease (CAD), whereas low scores do not rule out obstructive and high-risk plaques. A CAC score > 100 defines a category of patients with a risk comparable to patients with previous CAD [16]. Regarding luminal stenosis, a value > 50% is strongly associated with cardiovascular events [17]. Furthermore, by adding the affected segments, a segment involvement score is derived, providing a strong predictive measure for future cardiovascular events [18].

**Figure 1:**
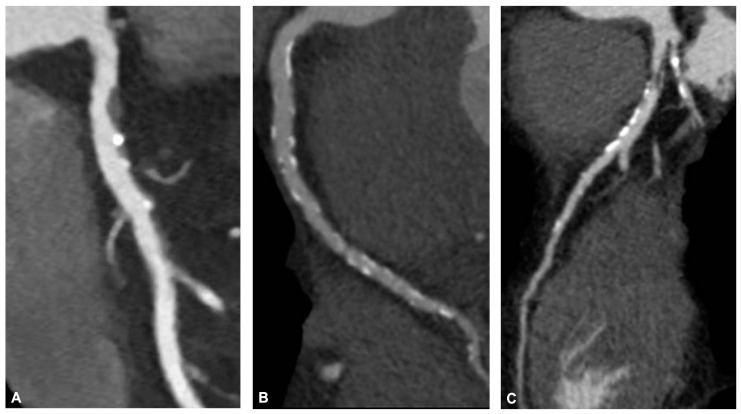
CCTA multiplanar reconstruction of coronary arteries in three master athletes. (**A**) Left anterior descendent artery of a 50-year-old golf player: the vessel shows a high-risk plaque in the proximal segment with a large soft component and a spotty calcification near the necrotic core. The maximum lumen stenosis is 60%. The patient underwent invasive functional assessment showing a significant FFR value (0.079) so that PCI and drug-eluting stent apposition were performed. (**B**) Right coronary artery of a 43-year-old cyclist: the vessel shows a diffuse calcific disease without significant stenosis. An optimization of medical therapy with statin was performed. (**C**) Left anterior descendent artery of a 48-year-old football player: CCTA highlights a diffuse atherosclerotic disease characterized by a significant stenosis of the proximal segment due to a mixed plaque with hypodense component and little calcifications. The middle part of the vessel shows a calcific coronary artery disease without relevant stenosis. The patient underwent PCI and stenting.

Further characteristics help to define high-risk plaques, such as a peculiar napkin ring sign, which indicates a plaque core that shows low CT attenuation surrounded by a rim-like area with higher Hounsfield units (HUs). Other relevant features include positive remodeling or vessel expansion, which can impact disease progression over time. Moreover, a low Hounsfield unit (below 30) denotes lipid enrichment, while spotty calcification can be useful as a potential marker for high-risk plaques [19]. Figure 1 shows three athletes with three different coronary plaques.

Data from the literature have pointed out that plaques among sportsmen are characterized by relatively different structures, with a higher likelihood of calcified plaques in athletes compared to sedentary individuals, where plaque composition is predominantly mixed [8,9]. In contrast, a recent multicenter prospective cohort study aimed to investigate the absolute prevalence of different coronary plaque types. It showed, for the first time, a tendency in sedentary individuals toward calcified plaques with smaller proportions of mixed and non-calcified plaques. Among 558 individuals, including 176 healthy non-athletes, 191 late-onset endurance athletes, and 191 lifelong endurance athletes, calcified plaques were accounted as the predominant type in both athletes and non-athletes, with mixed and non-calcified plaques following in prevalence. Interestingly, lifelong athletes exhibited: (i) a greater coronary plaque burden; (ii) a higher proportion of proximal plaques and lesions with substantial stenosis; (iii) plaques characterized by non-calcified and mixed morphology, which are more prone to rupture and potentially more unstable. Since the overall protective effect of physical activity on the progression of atherosclerosis is known, it has been hypothesized that lifelong middle-aged athletes’ overall plaque burden remains low, thanks to a lower plaque-to-vessel ratio, which can explain less significant stenoses during exercise [20]. To this mechanism, Aengevaeren V.L. et al. pointed out that very vigorous exercise could have the same effects as statins on plaque composition: on one hand, leading to increased calcification; on the other hand, mimicking the beneficial effect of statin, i.e., decreasing atheroma size and minimizing cardiovascular risk [21].

Several studies have also focused on the prevalence and composition of coronary plaques based on the type of sport practiced. In this context, it was proven that calcified plaques were more frequent in cyclists than in other types of sport, shedding light on the role of bone mineral density and parathyroid hormone, which could play a role in the process of atherosclerosis [11,12]. Additionally, endurance training appeared to be related to more non-calcified plaques in proximal segments than fit individuals with a similar risk profile [20].

**Figure 2:**
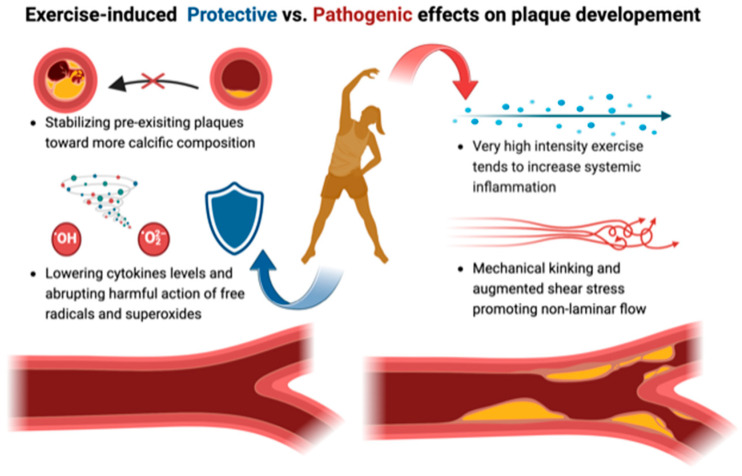
A schematic representation of possible mechanisms of the involvement of vigorous physical activity in CAD.

Concerning the mechanisms underlying the composition of plaques, the precise reasons for these observations in master athletes are still unclear. Factors that have been hypothesized include endothelial damage from exercise-induced shear stress, which may influence the flow patterns. These alterations could be caused or worsened by mechanical kinking of coronary arteries and exercise-induced spasm, as well as exercise-related hypertension [22]. Also, the generation of oxidative free radicals could play a role, with several studies reporting a correlation with the development of myocardial fibrosis and accelerated atherosclerosis: indeed, it appears that the protracted generation of oxidative stress with prolonged running is related to significant coronary obstructions [23]. Fascinating remains the involvement of systemic inflammatory response induced by repeated intensive exercise, which could be related to an acceleration in atherosclerosis development on one hand [24]. Figure 2 shows possible mechanisms of the involvement of vigorous physical activity on coronary plaques. On the other hand, animal studies have shown that exercise could potentially confer cardioprotective benefits by lowering levels of cytokines, reactive oxygen species, and superoxides [25].

## 4. Coronary Plaque Progression and Events in Athletes

Little is known about the progression of CAD (i.e., increase in CAC) among athletes. Aengevaeren and colleagues have recently demonstrated that there is no association between the volume of exercise and CAC or plaque progression in 318 participants of the Measuring Athlete’s Risk of Cardiovascular events (MARC) study, regardless of the amount of PA after a median 6-year follow-up [21]. These findings are in agreement with previous results observed in Norwegian runners in which the only factors associated with CAC progression were age and baseline CAC level [26]. Results from Aengevaeren’s study mainly concern the exercise intensity: the proportion of vigorous exercise on total exercise volume correlated negatively with progression of CAC, whereas very-high-intensity exercise was positively associated with it. Even though extremely high-intensity exercise was not associated with the absolute increase in number of coronary plaques, it was significantly associated with coronary plaque progression. Additionally, intensity of exercise was also related to development of CAC. It has been argued that the increased levels of circulating catecholamines—associated with high-to-very-high-intensity exercise—expose athletes to a higher risk of acceleration of atherosclerosis [21].

Although evidence is scarce in the field, some large epidemiological trials about the prevalence of atherosclerosis in athletes between the 1990s and 2000s have been published. In 2013, Delaney and colleagues stratified, according to the amount of physical activity, the Multi-Ethnic Study of Atherosclerosis (MESA) study participants who have had a follow-up CCTA scan some years after enrolment. The increase in CAC has been evaluated, and it has been shown that among athletes who already had coronary calcification at baseline, a lower degree of physical activity was strongly associated with the progression of CAC. Furthermore, vigorous physical activity was protective against the development of non-pre-existing CAC [5].

Conflicting results were obtained in two other analyses. In 2021, both Sung Y. et al. and Gao J.W. et al. stratified subjects from previous trials depending on their amount of PA and demonstrated greater progression at the CCTA follow-up in terms of CAC score in groups with higher levels of PA [27,28]. These latter data, confirming those obtained on populations of athletes only by Aengevaeren V.L. et al., would therefore indicate that a greater intensity of physical activity (measured in terms of MET/hours/week) could be associated not only with the presence of higher levels of CAC at baseline but also with their progression at follow-up [11].

These results appear necessarily in disagreement with those on the benefits of physical activity in delaying the process of atherosclerosis, demonstrated by solid scientific evidence [29]. However, this paradox seems to be resolved by data regarding cardiovascular prognosis of sportsmen with CAC, which—although numerically scarce—indicate that there is no increased risk of cardiovascular events or mortality from all causes in this population.

The first prognostic data were highlighted by Mohlenkamp’s study [6]. Cardiac magnetic resonance performed in addition to CAC quantification showed that 12% of the sample had ischemic-type late gadolinium enhancement (LGE) and that its presence was associated with higher CAC score values. The study also includes follow-up data (21 months) about cardiovascular events: no athlete died, but 4 of 108 subjects had coronary events (2 aborted cardiac arrests after PA; 1 acute myocardial infarction with ST-segment elevation; 1 surgical revascularization for CAD pointed out by additional testing), and 3 of these had LGE. Interesting data concern also the distribution of events according to CAC score values: no subject with CAC = 0 had events, while in CAC 100–400 and >400 groups, events concerned 8% and 14%, respectively, of the sample.

Looking strictly at cardiovascular events, Redford and colleagues demonstrated in 2018 that in an 8000-subject population, as CAC levels increased, an improvement in cardiorespiratory fitness (and consequently in the level of training) was associated with a lower risk of cardiovascular events [30]. Afterwards in 2021, Gao J.W. and colleagues confirmed previous evidence about an association between progression of CAC and increased risk of cardiovascular events in the general population, but they pointed out that the risk did not vary depending on the amount of physical activity [28].

**Figure 3:**
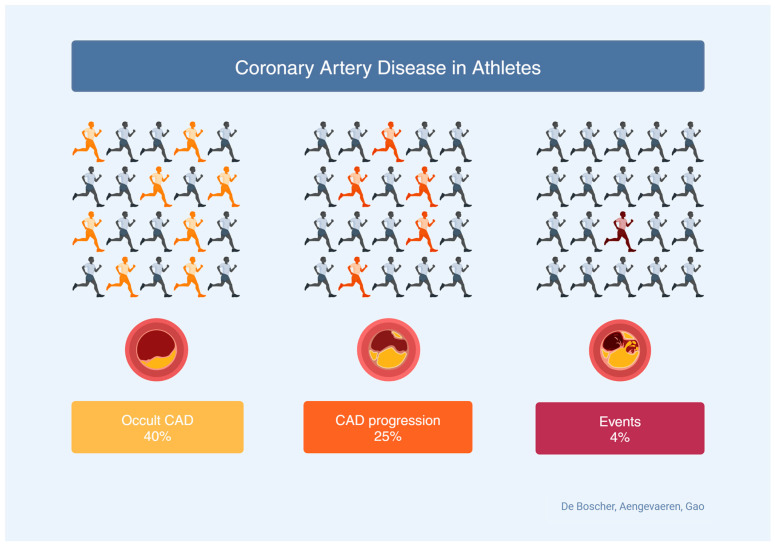
A summary of incidence, progression, and prognosis of CAD in athletes.

The prognosis of athletes with high levels of CAC is good even if all-cause mortality is considered. In 2017, Arnson Y. et al. divided 11,000 asymptomatic subjects referred for a screening CAC scanning into four groups according to physical activity level: among those with CAC = 0, there was no difference in terms of all-cause death, while among those with CAC > 0, mortality risk was inversely associated with the amount of physical activity, especially among those with a higher CAC score [31]. German C.A. and colleagues also confirmed these data in 2022, dividing the 6000 MESA trial subjects into two risk categories depending on CAC levels and stratifying them by level of physical activity: both in low-risk (CAC < 100) and high-risk subjects (CAC > 100), an increase in the amount of physical activity was protective against all-cause mortality [32]. Figure 3 summarizes the incidence, progression, and prognosis of CAD in athletes.

Regarding the prognosis of athletes with CAC, one missing piece of information is data about the use of percutaneous coronary intervention (PCI). Currently, the only available evidence is that of the MARC 2 trial in which 2 of 314 subjects had been sent to PCI [21]. However, it has to be taken into account two major limitations of the cited studies: first, they are mostly retrospective analyses, and second, the analyzed population did not include only athletes. So, it is currently hard to identify those belonging to this category with a univocal definition. Hence, it will be interesting to have the follow-up data from the Master@Heart study, which currently represents a unicum on this topic [33].

## 5. Conclusions and Future Directions

The present review summarizes current evidence about the intriguing dynamics of coronary artery plaques among athletes, offering insights into their composition, prevalence, and potential implications for cardiovascular health. Data from the literature underscore the complexity of atherosclerosis in this unique population and raise pertinent questions regarding the interplay between physical activity, plaque morphology, and cardiovascular risk. Table 1 summarizes all available data about coronary atherosclerosis in athletes.

One of the noteworthy observations is the relatively higher prevalence of calcified plaques among athletes compared to sedentary individuals. This finding challenges conventional assumptions about plaque composition in physically active individuals and suggests a potential protective effect of exercise against the development of non-calcified and mixed plaques. The predominance of calcified plaques in athletes may be attributed to various factors, including the impact of mechanical stress on coronary vasculature, adaptive responses to exercise-induced shear forces, and metabolic adaptations associated with long-term endurance training [22,23,24,25].

The implications of plaque composition extend beyond mere anatomical characterization, as they have a significant prognostic role. While calcified coronary plaques are generally considered more stable and less prone to rupture, mixed and non-calcified ones exhibit greater vulnerability, and they are associated with an increased risk of cardiovascular events [34]. Therefore, the predominance of calcified plaques among athletes may confer a degree of protection against adverse cardiovascular outcomes, although further research is needed to confirm and elucidate the underlying mechanisms.

Another notable finding is the association between coronary plaque morphology and exercise intensity, duration, and type. It has been revealed that athletes engaged in vigorous and prolonged exercise tend to exhibit a higher burden of coronary plaques, particularly those characterized by non-calcified and mixed morphology. This observation highlights the importance of considering the dose–response relationship between physical activity and cardiovascular health, as excessive exercise may paradoxically increase the risk of atherosclerosis and adverse cardiovascular events [21].

Furthermore, the potential role of coronary plaque composition as a marker of cardiovascular risk stratification in athletes has to be considered. Current guidelines enhance the role of CCTA, recommending its use in case of cardiovascular risk assessed by SCORE ≥ 5 and/or abnormal ergometric test [35]. Incorporating advanced imaging modalities such as CCTA into cardiovascular assessment protocols for athletes plays a key role in risk stratification and facilitates management strategies tailored to individual risk profiles. As regards cardiovascular prevention in this population, no particular management has been provided till now; educational programs seem to be the most useful approach in athletes who already conduct a good lifestyle.

Future research endeavors should focus on elucidating the mechanistic underpinnings of coronary plaque formation and progression in athletes, exploring the long-term effects of exercise on plaque stability, vulnerability, and related events, and evaluating the efficacy of targeted interventions aimed at mitigating cardiovascular risk in this population.

## Figures and Tables

**Table 1:** A summary of available data about coronary artery disease in athletes.

Study	AIM	Design and Methods	Results	Conclusions
Mohlenkamp 2008 [6]	Quantify the prevalence of CAC in relation to CV risk factors in marathon runners and study its role in myocardial damage and coronary events	108 marathon runners > 50 y with no CAD, angina, or DM	- high atherosclerosis in athletes - similar CAC in runners vs. ctrl (probably underestimated because of CV risks)	Elevated CAC may contribute to increased myocardial damage. Frequent marathon running may not protect these athletes from the risk of coronary events.
Schwartz 2014 [10]	CCTA to assess correlation between marathon running in men and quantitative plaque differences compared to sedentary	50 athl vs. 28 ctrl regardless of CV risks	Runners have increased total plaque volume, calcified plaque volume, and non-calcified plaque volume.	Long-term training may be paradoxically associated with accelerated coronary artery plaque formation.
Braber et al. MARC 2016 [7]	Value of CCT (CCTA+CACS) to detect occult CAD in asymptomatic men patients (94% low risk according to ESC score1)	Prospective - sup>- cut-off values CACS > 100 and >50% stenosis ^1^	- combination of CACS and CCTA identified CAD in 60 individuals - NNS of 159 - NNT 30	CACS could help to prevent cardiac events and be cost-effective compared to exercise testing.
Aengevaeren 2017 [8]	Determine the relationship between lifelong exercise volumes and coronary atherosclerosis	Analysis of the MARC-1 study	- no difference in CAC area, density, and number of lesions among exercise volume groups in those with CAC - higher plaque prevalence in the most active group versus the least active group - lower prevalence of mixed plaques was observed in the most active vs. least active group - the most active group significantly more often had only calcified plaques compared with the least active group	Beneficial vascular adaptations such as an improved coronary flow reserve may also allow athletes to better deal with coronary stenoses and to experience fewer symptoms and events than the general population with a similar plaque burden.
Merghani 2017 [9]	Prevalence of subclinical CAD in Masters Endurance athletes	152 athletes including runners and cyclists with more strictly inclusion criteria according to risk factors	- higher CACS and higher number of plaques - no relationship dose exercise and calcifications	Higher CACS in athletes with CAC > 0 compared with ctrls with CACS > 0 in contrast with Aengevareen and MohlenkampI
Aengevaeren V.L., 2019 [11]	Assess impact of different sports on plaque prevalence and plaque morphology	Post hoc analysis of the MARC	- Cyclists had a lower prevalence of plaques and lower prevalence of CAC compared with runners. - Cyclists had more calcified plaques.	- Role of bone turnover? - Role of PTH? - Lower intensity in cycling?
Aengevaeren 2023 MARC-2 analysis [21]	Evaluate progression of coronary atherosclerosis in 289 male athletes using CAC scoring and CCTA and assessed its association with PA characteristics during 6.3 y follow-up	Analysis of the MARC-2		- Ex. intensity but not volume associated with progression of CAD - Vigorous PA = less CAC progression - Very vigorous PA = greater CAC progression
De Bosscher 2023 [20]	The primary endpoint was the prevalence of coronary plaques on CTCA assessed cross-sectionally at baseline. Hypothesized that lifelong endurance exercise would be associated with a lower prevalence of non-calcified plaques than non-athletes.	Prospective observational cohort study	- First to investigate the absolute prevalence of different coronary plaque types - lifelong middle-aged athletes had more coronary plaques, including more unstable non-calcified plaques in proximal segments.	- lower risk of CV events amongst fit individuals not explained by plaques composition/extent - overall plaque burden remains low in athletes - plaque-to-vessel ratio in athletes lower = less significant stenosis
Roberts 2017 [14]	CCTA and cardiac risk factor in women with long-term marathon running histories to compare to sedentary women with similar risk factors.	Observational on 56 women	- Runners had lower CACC, had lower calcified plaque volumes, were leaner, and smoked tobacco less compared with sedentary.	The development of coronary plaque in women marathon runners appears to be related to older age and numerous cardiovascular risk factors

^1^ CAD defined as CACS 100 Agatston units (AU) on non-contrast CCT and/or 50% luminal stenosis on CCTA.
